# Integrative analysis of DNA methylation and gene expression to identify key epigenetic genes in glioblastoma

**DOI:** 10.18632/aging.102139

**Published:** 2019-08-08

**Authors:** Danyun Jia, Wei Lin, Hongli Tang, Yifan Cheng, Kaiwei Xu, Yanshu He, Wujun Geng, Qinxue Dai

**Affiliations:** 1 Department of Anesthesiology, The First Affiliated Hospital of Wenzhou Medical University, Wenzhou 325000, Zhejiang, China; 2 Department of Pediatric Intensive Care Unit, The Second Affiliated Hospital and Yuying Children's Hospital of Wenzhou Medical University, Wenzhou 325027, Zhejiang, China; 3 Department of Neurology, The First Affiliated Hospital of Wenzhou Medical University, Wenzhou 325000, Zhejiang, China

**Keywords:** glioblastoma, TCGA, methylation, gene expression, biomarker

## Abstract

Glioblastoma (GBM) ranks the most common and aggressive primary brain malignant tumor worldwide. However, the survival rates of patients remain very poor. Therefore, molecular oncology of GBM are urgently needed. In this study, we performed an integrative analysis of DNA methylation and gene expression to identify key epigenetic genes in GBM. The methylation and gene expression of GBM patients in The Cancer Genome Atlas (TCGA) database were downloaded. After data preprocessing, we identified 4,881 differentially expressed genes (DEGs) between tumor and normal samples, including 1,111 upregulated and 3,770 downregulated genes. Then, we randomly separated all samples into training set (n = 69) and testing set (n = 69). We next obtained 11,269 survival-methylation sites by univariate and multivariate Cox regression analyses. In the correlation analysis, we defined 198 low promoter methylation with high gene expression as epigenetically induced (EI) genes and 111 high promoter methylation with low gene expression as epigenetically suppressed (ES) genes. Key markers including C1orf61 and FAM50B were selected with a Pearson correlation coefficient greater than 0.75. Further, we chose the 20 CpG methylation sites of above two genes in unsupervised clustering analysis using the Euclidean distance. We found that the prognosis of the hypomethylated group was significantly better than that in the hypermethylated group (log-rank test *p*-value = 0.011). Based on the validation in the TCGA testing set and GEO dataset, we validated the prognostic value of our signature (*p*-value = 0.02 in TCGA and 0.012 in GEO). In conclusion, our findings provided predictive and prognostic value as methylation-based biomarkers for the diagnosis and treatment of GBM.

## INTRODUCTION

Glioblastoma (GBM) ranks the most common and aggressive primary brain tumor worldwide [1]. It is a fast-growing malignant tumor that arises from multiple cell types with neural stem-cell-like properties. Besides conventional therapy, current approaches such as small molecules and gene therapy are developed in recent years [2, 3]. New synthetic small molecules were discovered as promising anti-GBM agents [3]. Although with various treatments, patient outcomes remain between 12 and 15 months survival rate, and with five-year survival rates at only 10% [4]. Therefore, advances in the field of molecular oncology of GBM are urgently needed.

The major factors contributing to the pathogenesis of human cancers were epigenetic molecular mechanisms, including GBM [5]. With the help of gene microarray and RNA-seq, the aberrant expression profiles of GBM in genome and transcriptome level were increasing reported. Using the gene expression data from Gene Expression Omnibus (GEO) database, Bo et al. [6] identified a total of 431 differentially expressed genes (DEGs) between GBM and normal samples. After various bioinformatics analysis, 69 DEGs were identified significantly associated with GBM prognosis. Another study found 486 DEGs based on the gene expression profile of GSE50161 [7]. CDK1, CCNB1 and CDC20 were selected in survival analysis and high expression was significantly associated with poor survival in GBM. However, numerous identified DEGs will not contribute to the clear understandings of biological pathogenesis of GBM.

DNA methylation was found in the dinucleotides of nearly eighty percent of the CpG islands in the genome [8]. It was catalyzed by DNA methyltransferases that controls various cell activities such as proliferation, apoptosis, and differentiation. As for human cancers, methylation was known to be abnormal in all forms of cancers [9] and abnormal methylation of promoters could lead to silence of tumor suppressor genes, affecting transcriptional pathways and resulting in the cancer development [10]. In addition, targeted drugs about DNA methyltransferase inhibitors have been approved for the treatment of chronic myelomonocytic leukemia and acute myelogenous leukemia as well as a second generation of DNA methyltransferase inhibitors [11]. Intra-tumor DNA methylation heterogeneity has been proved a feature of GBM [12]. Moreover, the promoter methylation status of the O^6^-methylguanine-DNA methyltransferase (MGMT) gene has been described as the predictor of chemotherapeutic response and patients’ survival in GBM [13]. Wang et al. [14] developed a signature with three genes (FPR3, IKBIP and S100A9) signature for prognosis in patients with MGMT promoter-methylated GBM using data from Chinese Glioma Genome Atlas (CGGA) and TCGA. In another study, Wen et al. [15] performed analysis of methylated genes as potential biomarkers in evaluating malignant degree of GBM. In this study, they found a total of 668, 412, 470, and 620 methylation or demethylation genes associated with the degree of GBM from grades 1 to 4. Therefore, abnormal methylation genes can act as potential oncogenes or anti-oncogenes in the development and progression of cancers, suggesting their potential roles as biomarkers.

In the present study, we performed an integrative analysis of DNA methylation and gene expression identified key epigenetic genes in GBM. The methylation and gene expression of GBM patients in TCGA database were downloaded. After data preprocessing, we identified 4,881 DEGs between tumor and normal samples, including 1,111 upregulated and 3,770 downregulated genes. Then, we randomly separated all samples into training set and testing set. We next obtained 11,269 survival-methylation sites by univariate and multivariate Cox regression analyses. In the correlation analysis, we defined 198 low promoter methylation with high gene expression as EI genes and 111 high promoter methylation with low gene expression as ES genes. Key markers including C1orf61 and FAM50B were selected with a Pearson correlation coefficient greater than 0.75. Further, we chose the 20 CpG methylation sites of above two genes in unsupervised clustering analysis using the Euclidean distance. We found that the prognosis of the hypomethylated group was significantly better than that in the hypermethylated group (log-rank test *p*-value = 0.011). Based on the validation in the TCGA testing set and GEO dataset, we validated the prognostic value of our signature (*p*-value = 0.02 in TCGA and 0.012 in GEO). In conclusion, our findings provided predictive and prognostic value as methylation-based biomarkers for the diagnosis and treatment of GBM.

## RESULTS

### DNA methylation data selection and characteristics

**Figure 1:**
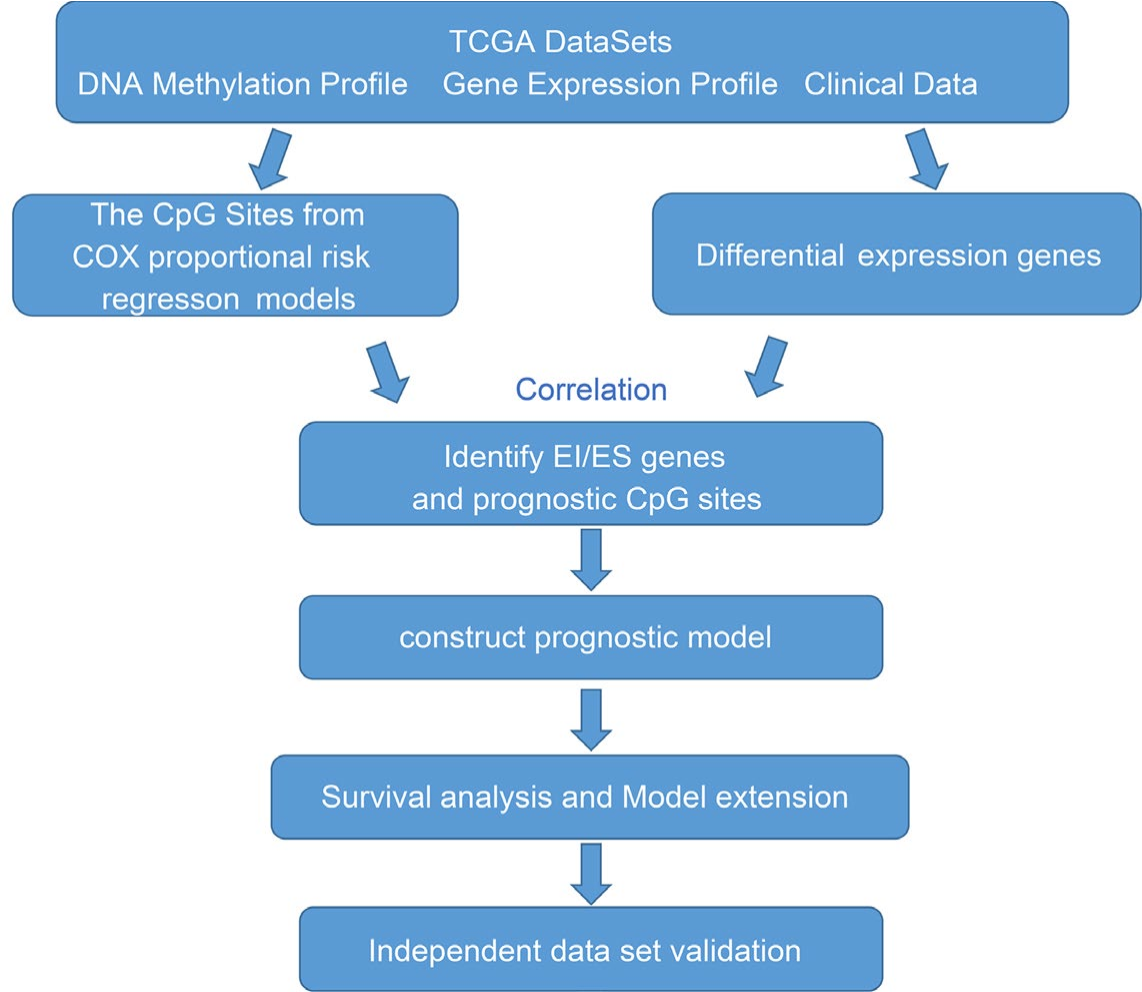
**The workflow of the present study.**

In this study, we performed an integrative analysis of DNA methylation and gene expression identified key epigenetic genes in GBM (Figure 1). We used the gene expression and DNA methylation profiles from TCGA database. A total of 138 GBMs and normal samples with clinical information data were obtained. Moreover, there were 20,530 genes were downloaded from the TCGA database for subsequent analysis. Because DNA methylation in promoter regions strongly influences gene expression, we selected CpGs in promotor regions that were defined as 2 kb upstream to 0.5 kb downstream from TSS. After preprocessing data, we finally obtained 145,907 methylation sites for downstream analysis.

### Clinical patient characteristics

We obtained the clinical information including sample ID, vital status, age at initial pathologic diagnosis, days to death, days to last follow up, and grade. All samples were randomly divided into two groups: the training set (n = 69) and the testing set (n = 69). The training set and test set are required to meet the following criteria: first, samples are randomly assigned to training set and testing set; second, the age distribution, follow-up time and patient death rate should be similar in these two groups. The expression profiles and clinical information of training set were shown in Supplementary Tables 1 and 2, respectively. In addition, the expression profiles and clinical information of testing set were shown in Supplementary Tables 3 and 4, respectively.

### Determining DEGs of GBM

**Figure 2:**
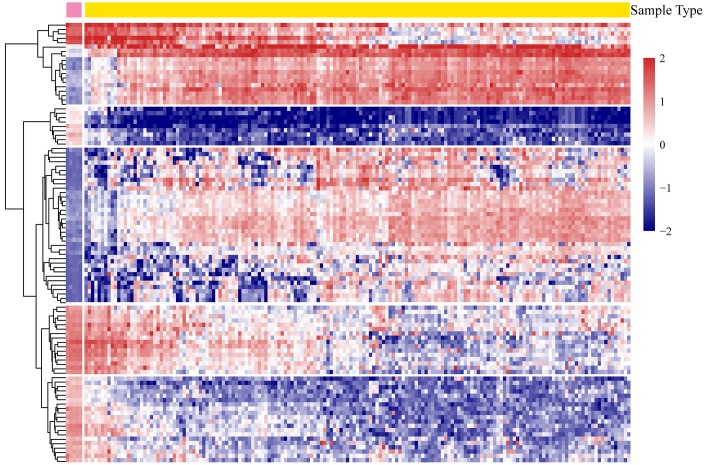
**The heatmap expression profiles of the most significant 100 genes.**

According to the screening criteria, a total of 4,881 significant DEGs were obtained from all the tumor and normal samples (Supplementary Table 5). There were 1,111 genes were upregulated and 3,770 genes downregulated. The expression profiles of the most significant 100 genes were shown in Figure 2.

### Survival analysis of methylation sites in the training set

In order to determine methylation sites associated with survival outcomes, we performed univariate and multivariate Cox regression analyses of the obtained methylation sites of GBM. There were a total of 11,269 methylation sites and we generated a new survival-methylation expression profiles for further analysis (Supplementary Table 6).

### Correlation analysis of DEGs and survival-methylated genes

DNA methylation level can affect the gene expression. High methylation expression often inhibits downstream gene expression, and low methylation level tends to increase the downstream gene expression. The correlation analysis steps for calculating differentially expressed genes and differentially methylated genes were as follows: 1) Calculating the intersection of differentially methylated genes and DEGs. 2) Identifying the number of genes whose differential expression was up-regulated and differentially methylated was down-regulated. In addition, identifying the number of genes whose differential expression was down-regulated and differential methylation was up-regulated. Therefore, a total of 324 up-regulated genes, 162 down-regulated genes, 249 methylated down-regulated genes, and 237 methylated up-regulated genes were obtained (Supplementary Table 7).

**Figure 3:**
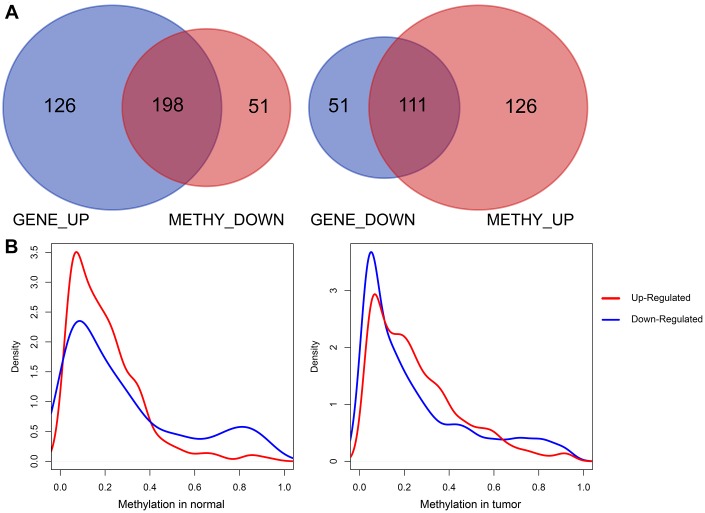
**Correlation analysis of DEGs and survival-methylated genes.** (**A**) The intersection results of DEGs and survival-methylated genes. (**B**) Distribution of promoter methylation levels in tumor and normal samples.

We then analyzed the Pearson correlations between upregulated DEGs and downregulated survival-methylated genes, as well as downregulated DEGs and upregulated survival-methylated genes. As shown in Figure 3A, we found that there were a total of 198 genes between upregulated DEGs and downregulated survival-methylated genes. In addition, 111 genes were selected between downregulated DEGs and upregulated survival-methylated genes. Next, we performed analysis of the promoter methylation distribution of DEGs between tumor samples and normal samples. The results showed that highly expressed genes in tumors had lower promoter methylation in normal samples, indicating a negative correlation between promoter DNA methylation and gene expression in normal and tumor tissues (Figure 3B).

### Pathway enrichment analysis of EI and ES genes

We found a total of 198 low promoter methylation with high gene expression (EI genes), as well as a total of 111 high promoter methylation with low gene expression (ES genes) (Supplementary Table 8).

**Figure 4:**
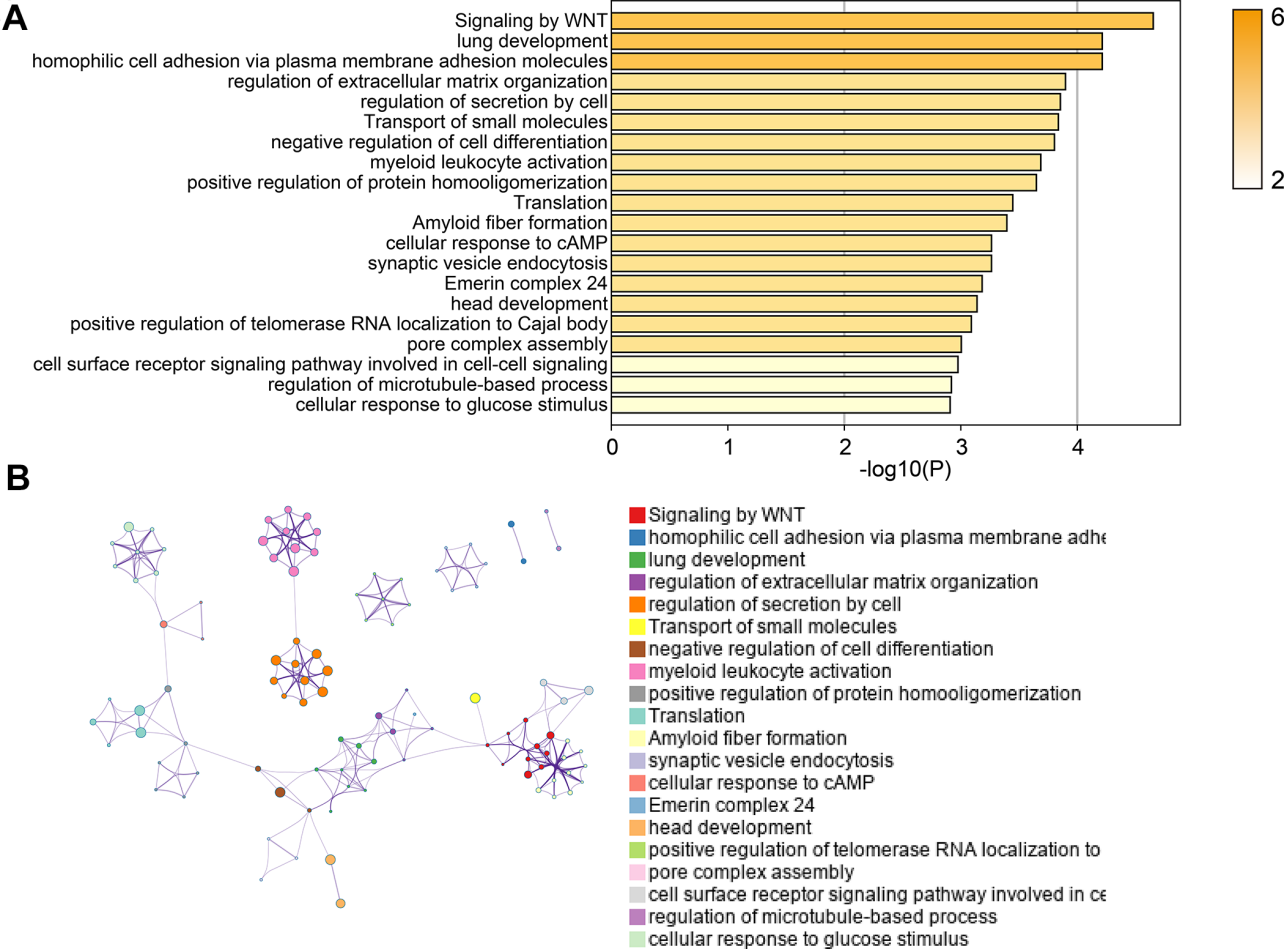
**Pathway enrichment analysis of EI and ES genes.** (**A**) The pathway enrichment results of EI and ES genes. (**B**) The network diagram of interacting genes.

Next, we used online tools “Metascape” to performed pathway enrichment analysis. As shown in Figure 4A, we found that EI and ES genes were significantly enriched in pathways including Signaling by WNT, negative regulation of cell differentiation, regulation of extracellular matrix organization, and cellular response to cAMP. The “Metascape” also provided the interactions of genes based on these pathways (Figure 4B). These results suggested that EI and ES genes screened in our study were involved in the biological process of the occurrence and development of GBM.

### Construction of the prognosis risk model based on methylation genes

In order to further screen potential EI and ES genes, Pearson correlation analysis was used to calculate the correlation between promoter methylation and gene expression of EI and ES genes. There were 16 key genes with negative correlations. Next, we selected genes with a correlation coefficient greater than 0.75 as key markers. They were C1orf61 and FAM50B.

**Figure 5:**
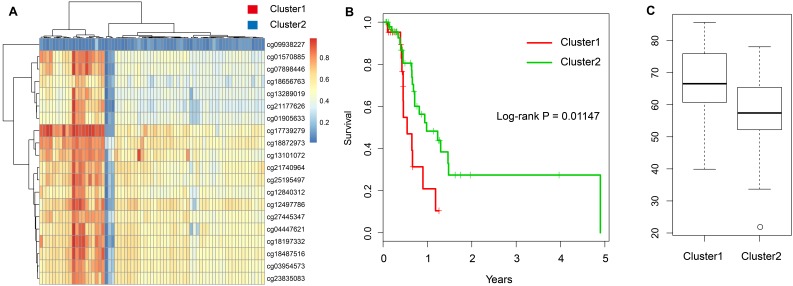
**Construction of the prognosis risk model based on methylation genes.** (**A**) The heatmap of 20 methylation sites in the training set. (**B**) The K-M plot of the hypomethylated and hypermethylated groups. (**C**) The age distribution of patients in the hypomethylated and hypermethylated groups.

Further, we chose the 20 CpG methylation sites of above two genes (Table 1) in unsupervised clustering analysis. Using the Euclidean distance to calculate the similarity between samples, we found that all samples can be divided into two groups Cluster 1 and Cluster 2 according to the 20 CpG methylation sites. Moreover, the samples in Cluster 1 were with high methylation level, but samples in Cluster 2 were with low methylation level (Figure 5A). Further analysis was performed to explore the prognosis between two groups. As shown in Figure 5B, we found that the prognosis of the hypomethylated group was significantly better than that in the hypermethylated group (log-rank test *p*-value = 0.011). Moreover, we compared the ages of patients in these two groups and found that the age distribution of patients in the hypomethylated group was lower than that in the hypermethylated group (Figure 5C).

**Table 1:** The annotation of 20 CpG sites.

**cg probe**	**Gene**	**Chrom**	**Site**
cg09938227	C1orf61	1	156390124
cg18197332	FAM50B	6	3849458
cg01570885	FAM50B	6	3849272
cg04447621	FAM50B	6	3849475
cg21740964	FAM50B	6	3849331
cg07898446	FAM50B	6	3849294
cg18487516	FAM50B	6	3849542
cg18872973	FAM50B	6	3849095
cg25195497	FAM50B	6	3849327
cg13101072	FAM50B	6	3849818
cg21177626	FAM50B	6	3849411
cg18656763	FAM50B	6	3849235
cg27445347	FAM50B	6	3849801
cg03954573	FAM50B	6	3849434
cg01905633	FAM50B	6	3849391
cg23835083	FAM50B	6	3849536
cg12840312	FAM50B	6	3849381
cg12497786	FAM50B	6	3849577
cg13289019	FAM50B	6	3849350
cg17739279	FAM50B	6	3849190

### IDH1 mutation and DNA methylation in GBM

**Figure 6:**
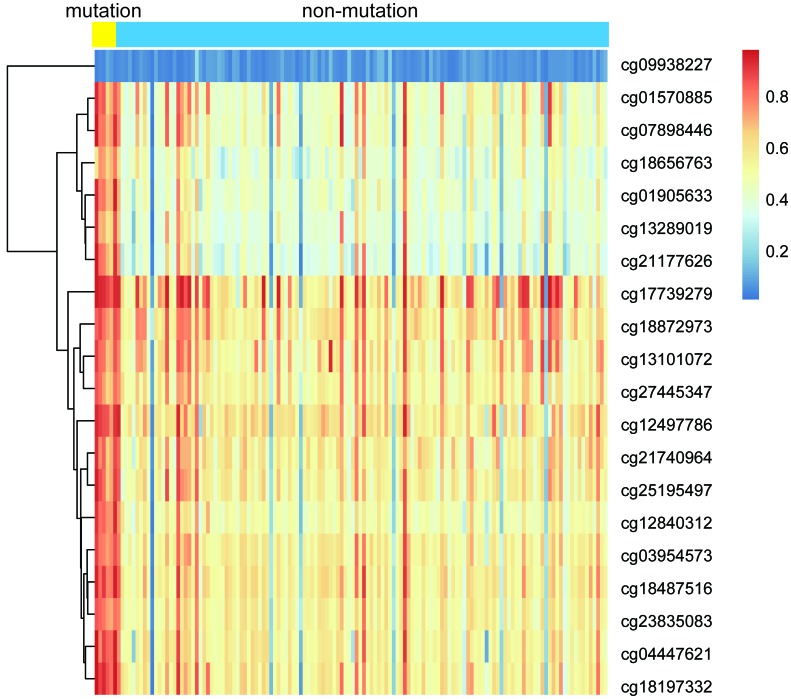
**The heatmap of IDH1 mutation and DNA methylation in GBM.**

IDH mutation is a phenomenon that occurs in the early stage of tumor and IDH mutation is considered as an important marker of low-grade glioma and GBM. IDH mutation can promote the hypermethylation of CpG in the promoter of most genes which contributes to the epigenetic instability of tumor cells. To explore the association between IDH1 mutation and DNA methylation in GBM, all samples were divided into IDH mutation group (n = 7) and IDH non-mutation group (n = 131) according to the IDH1 gene mutation. As shown in Figure 6, samples in IDH mutation group exhibited lower methylation level than that in IDH non-mutation group.

**Figure 7:**
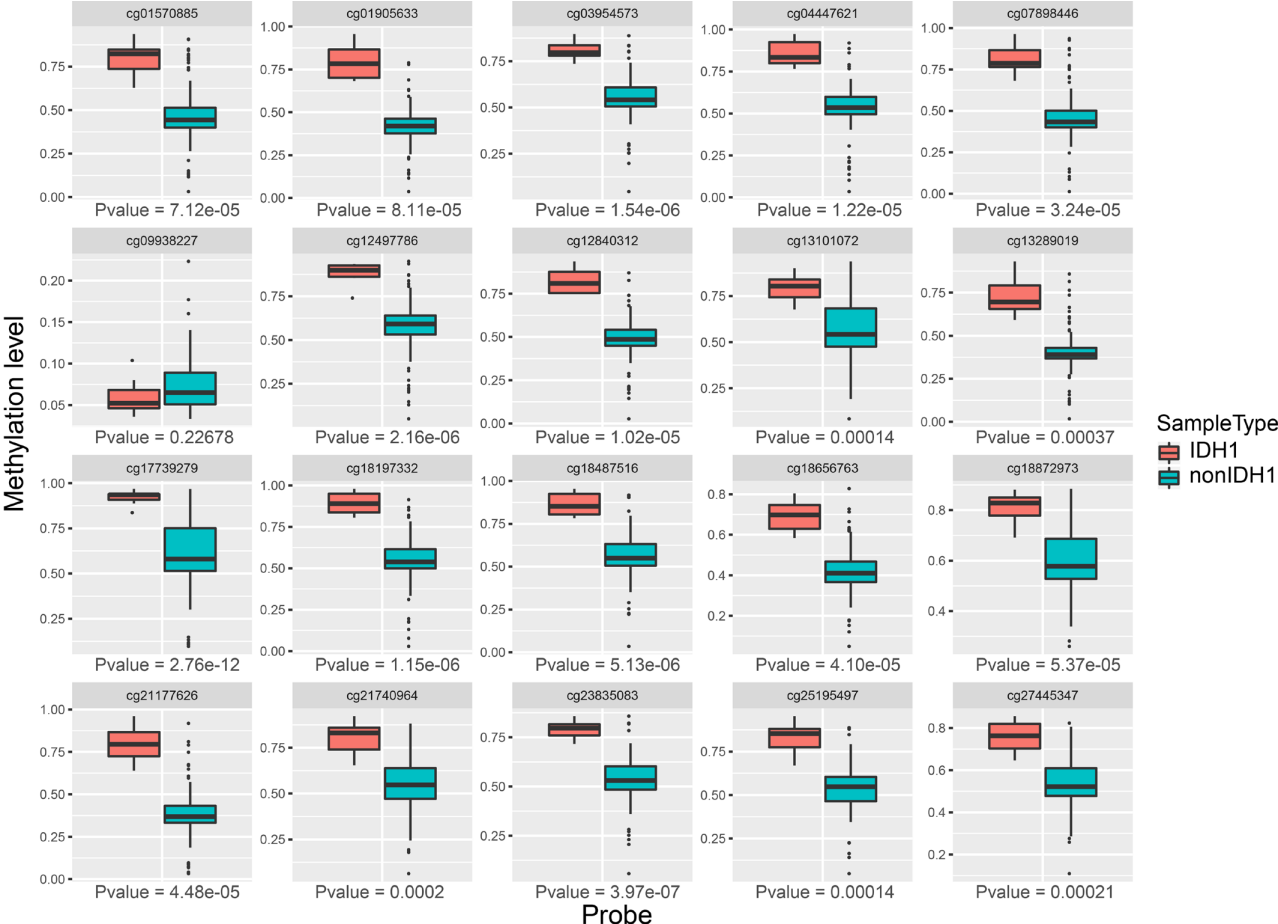
**The expression profiles of 20 methylation sites between IDH1 mutation and non-mutation groups.**

Then, we compared the expression of each methylation site in two groups. As shown in Figure 7, we found that 19 of the 20 sites were significantly expressed between IDH mutation and IDH non-mutation groups (*p*-value < 0.01). Above results suggested that these methylation sites were closely associated with IDH1 mutation.

### Validation in the TCGA testing set and GEO dataset

**Figure 8:**
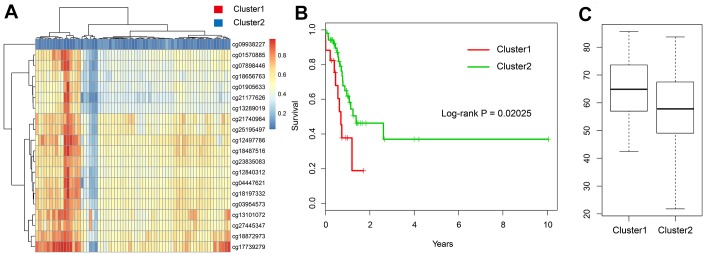
**Validation in the TCGA testing set.** (**A**) The heatmap of 20 methylation sites in the testing set. (**B**) The K-M plot of the hypomethylated and hypermethylated groups in the testing set. (**C**) The age distribution of patients in the hypomethylated and hypermethylated groups in the testing set.

To validate the results of our methylation data and prognostic model, we used the testing set (n = 69) based on TCGA data. We used the expression of 20 methylation sites and further used hierarchical cluster analysis. We found that the 20 CpG methylation sites can also clearly divide all samples into two groups (Figure 8A). The methylation levels of Cluster 1 group were significantly higher than Cluster 2. Moreover, the prognosis of samples in the hypomethylated group was significantly better than that in the hypermethylated group (log-rank test *p*-value = 0.02) (Figure 8B). It can also be seen that the age distribution in hypomethylated group was lower than that in the hypermethylated group, which was consistent with the results of the training set (Figure 8C).

**Figure 9:**
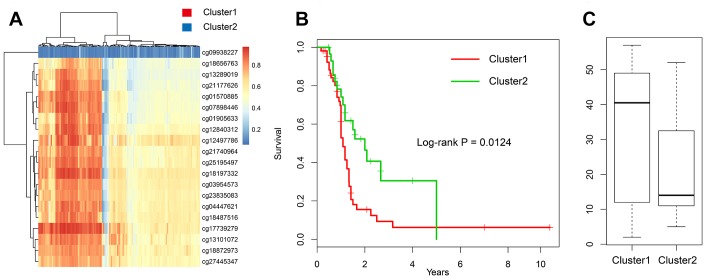
**Validation in the GEO dataset.** (**A**) The heatmap of 20 methylation sites in GSE36278. (**B**) The K-M plot of the hypomethylated and hypermethylated groups in GSE36278. (**C**) The age distribution of patients in the hypomethylated and hypermethylated groups in GSE36278.

In addition, the DNA methylation (GSE36278) [16] of GBM was downloaded with a total of 142 patients. First, we selected the expression profiles of 20 methylation sites (Supplementary Table 9) and clinical information (Supplementary Table 10). Next, we divided all samples into two groups using hierarchical cluster method (Figure 9A). Results showed that significant survival difference was found in two groups (log-rank test *p*-value = 0.012) (Figure 9B). Moreover, we compared the age distribution between two groups and found that high methylation group was higher than low methylation group (Figure 9C). These results were consistent with TCGA dataset, suggesting that this model can be applied to other samples.

## DISCUSSION

In the present study, we performed an integrative analysis of DNA methylation and gene expression identified key epigenetic genes in GBM. We obtained 11,269 survival-methylation sites by univariate and multivariate Cox regression analyses. In the correlation analysis, we defined 198 low promoter methylation with high gene expression as EI genes and 111 high promoter methylation with low gene expression as ES genes. Further, we chose the 20 CpG methylation sites of above two genes in unsupervised clustering analysis using the Euclidean distance. We found that the prognosis of the hypomethylated group was significantly better than that in the hypermethylated group. Based on the validation in the TCGA testing set and GEO dataset, we validated the prognostic value of our signature. Our findings provided predictive and prognostic value as methylation-based biomarkers for the diagnosis and treatment of GBM.

The occurrence and proliferation of cancer is regulated by epigenetic and genetic events, as well as epigenetic modifications. They are increasingly identified as important targets for cancer research [10]. DNA methylation catalyzed by DNA methyltransferases (DNMTs) is one of the important epigenetic mechanisms for controlling cell proliferation, apoptosis, differentiation, cell cycle and transformation in eukaryotes. Abnormal DNA methylation in cancer can be produced by mutation before or after cell transformation [9]. Moreover, it can regulate normal gene expression and facilitate chromatin organization within cells, which are accompanied by alterations in chromatin structure at gene regulatory regions [17]. Also, there were many literatures about the use of DNA methylation measurements for cancer diagnosis through examples of methylated genes [18].

In GBM, there are several studies about the molecular roles of DNA methylation. For examples, Wang et al. [19] used the gene expression and methylation profiles from TCGA as well as the Chinese Glioma Genome Atlas (CGGA) database. A total of 3,365 DEGs were identified with 2,940 genes expressed hypomethylation and high expression, while 425 genes showed hypermethylation and low expression in GBM. The eight genes (C9orf64, OSMR, MDK, MARVELD1, PTRF, MYD88, BIRC3, RPP25) were characterized to divide GBM patients into two groups with different survival outcomes. In addition, different clinical and molecular characteristics were also shown between the two groups. In another study, the positive prognostic value of MGMT promoter hypermethylation has been demonstrated in adult GBM, and the MGMT promoter methylation status is a clinically relevant predictor of the newly diagnosed GBM elderly population [19]. The roles of MGMT promoter methylation in GBM were also reported in various studies [20–22]. Ma et al. [23] reported that the hypermethylation of CXCR4 can predict patients’ OS in GBM. Besides, the methylation of AURKA, KIF4A, and NUSAP1 in GBM was also investigated [24].

In our study, key markers including C1orf61 and FAM50B were selected with a Pearson correlation coefficient greater than 0.75. Chromosome 1 open reading frame 61 (C1orf61) was reported to be up-regulated in hepatic cirrhosis tissues and up-regulated in primary hepatocellular carcinoma. Moreover, hepatitis B virus (HBV)-positive patients exhibited significantly higher levels of C1orf61 expression than HBV-negative patients. The overexpression of C1orf61 promoted cell proliferation and colony formation, as well as cell cycle progression. In addition, the overexpression of C1orf61 facilitated cellular invasion and metastasis. The overexpression of C1orf61 induced the epithelial-mesenchymal transition (EMT) that is linked to metastasis [25]. FAM50B (family with sequence similarity 50, member B) was shown that average methylation level of FAM50B was lower in asthenozoospermia group than in control group [26]. CpG sites (mapped to gene FAM50B) were also reported to be differentially expressed in the study of 24-hour exposure to air pollution [27]. DNA methylation changes of FAM50B in individuals with developmental delay/intellectual disability were observed [28]. However, these two genes were not reported in GBM.

DNA methylation patterns can predict prognosis and survival of human cancers [29]. The utility of methylation biomarkers for the molecular characterization of cancer with implications for patients’ prognosis. In one study, researchers identified and validated biomarkers for melanoma development (HOXA9 DNA methylation) and tumor progression (TBC1D16 DNA methylation). In addition, this study determined a prognostic signature with potential clinical value [30]. Gastric cancers showed significantly lower LINE-1 methylation levels compared to matched normal gastric mucosa and hypomethylation of LINE-1 was significantly associated with shorter overall survival [31]. Moreover, in the study of esophageal squamous cell carcinoma, LINE-1 hypomethylation is associated with a poor prognosis among patients [32]. Its methylation level was also associated with hepatocellular carcinomas [33]. Based on TCGA methylation expression profiles of gastric cancer, Hu et al. performed a DNA methylation gene signature consisting of five genes (SERPINA3, AP000357.4, GZMA, AC004702.2, and GREB1L) [34]. In addition, in other human cancers, there were also various studies about DNA methylation and prognostic signature, such as head and neck squamous cell carcinoma [35], cutaneous melanoma [36], glioma [37], and lung cancer [38]. Above results suggested that significant DNA methylation genes may be a new predictor and prognostic biomarker for cancers.

The prognostic ability of this methylation signature may improve the risk stratification of patients with GBM. In the future clinical application, this methylation signature may help people accurately guide clinical treatments and determine prognosis of patients. However, whether this signature can improve GBM diagnosis or treatment, it still remains unknown and this is what we will study in our future work. Besides, considering that C1orf61 was closely associated with cell proliferation, colony formation, cell cycle progression, and EMT, we assumed that this gene can participate in the occurrence and development of GBM through the above pathways. However, how methylation impacts the two key genes and their downstream effects are still the work we need to explore in the future.

In our study, we established a prognosis risk model based on methylation genes in GBM using the 20 CpG methylation sites of above two genes for GBM. In conclusion, our findings provided predictive and prognostic value as methylation-based biomarkers for the diagnosis and treatment of GBM.

## MATERIALS AND METHODS

### Data selection from TCGA database and preprocessing

All data were downloaded from the TCGA database (https://cancergenome.nih.gov/) [39] based on RNA-seq including DNA methylation, gene expression and IDH1 mutation expression profiles. The methylation data generated with the Illumina Infinium HumanMethylation 450 BeadChip array. The methylation level of each probe was represented by the β-value (from 0 to 1). First, the CpG sites with missing value > 70% of all samples were removed. Then, we used *impute* R package by k-nearest neighbors (KNN) method for the missing values of methylation data. We further removed the genomic unstable sites including CpGs in sex chromosomes and single nucleotide polymorphisms. We selected CpGs in promotor regions, which were defined as 2 kb upstream to 0.5 kb downstream from transcription start sites (TSS) [40]. Finally, we selected samples with gene expression profiles including a total of 138 tumor and normal samples.

All samples were separated into two cohorts: a training set (n = 69) and a testing set (n = 69). The methylation data of training set and clinical information (survival status, time, and age) was used to select CpG sites with prognostic value by univariate and multivariate COX proportional risk regression models. Last, according to the relationship between CpG sites and genes, we obtained key genes that significantly associated with survival.

### Determining DEGs of GBM and methylated sites

We used paired T-test as a statistical method to screen DEGs and methylated sites between tumor and normal samples, and multiple tests were performed for *p*-value correction. Finally, genes with false discovery rate (FDR) < 0.01 were screened as significant DEGs and methylated sites.

### Correlation analysis of DEGs and survival-methylated genes

To explore the association between DEGs and methylation, first, we used univariate Cox proportional risk regression model to analyze each methylation site and survival data. Then, clinical factors including grade and age were added as covariables for multivariate Cox regression analyses. Finally, the intersection results of univariate and multivariate Cox regression (*p*-value < 0.05) were obtained. Here, we defined genes with downregulated methylation in promoter region as downregulated survival-methylated genes, and genes with upregulated methylation in promoter region as upregulated survival-methylated genes.

We next performed the correlation analysis between upregulated DEGs and downregulated survival-methylated genes, as well as downregulated DEGs and upregulated survival-methylated genes. We used *Venny* software to screen the intersected genes. The average expression level of all methylated sites associated with survival represented the final expression level of this survival-methylated gene.

### Pathway enrichment analysis of epigenetically induced and epigenetically suppressed genes

In order to further identify the mutex genes, we defined low promoter methylation with high gene expression as EI genes. High promoter methylation with low gene expression as ES genes. Then, we used online tools “Metascape” (http://metascape.org) to performed pathway enrichment analysis of EI and ES genes.

### Construction of the prognosis risk model based on methylation genes

In order to further screen potential EI and ES genes, Pearson correlation analysis was used to calculate the correlation between promoter methylation and gene expression of EI and ES genes. We selected genes with a correlation coefficient greater than 0.75 as key markers. Hierarchical clustering algorithm was used to cluster the samples of the training set, and Euclidean distance was used to calculate the similarity between the samples. We used *survival* R package to observe whether the survival difference between the high-risk and low-risk groups by K-M survival analysis.

### IDH1 mutation and DNA methylation in GBM

To explore the association between IDH1 mutation and DNA methylation in GBM, according to the IDH1 gene mutation, all samples were divided into IDH mutation group (n = 7) and IDH non-mutation group (n = 131). 20 methylation sites were used to compare methylation differences between two groups.

### Validation in the TCGA testing set and GEO dataset

To validate the results of our methylation data and prognostic model, we used the testing set (n = 69) based on TCGA data. In addition, the DNA methylation (GSE36278) [18] of GBM was downloaded from NCBI GEO database (https://www.ncbi.nlm.nih.gov/geo/). A total of 142 patients with DNA methylation profiling were included for further validation. This dataset was carried on Illumina HumanMethylation450 BeadChip platform.

## Supplementary Material

Supplementary Table 1

Supplementary Table 2

Supplementary Table 3

Supplementary Table 4

Supplementary Table 5

Supplementary Table 6

Supplementary Table 7

Supplementary Table 8

Supplementary Table 9

Supplementary Table 10
